# Formation of Pt–Zn Alloy Nanoparticles by Electron-Beam Irradiation of Wurtzite ZnO in the TEM

**DOI:** 10.1186/s11671-016-1555-x

**Published:** 2016-07-20

**Authors:** Sung Bo Lee, Jucheol Park, Peter A. van Aken

**Affiliations:** Department of Materials Science and Engineering and Research Institute of Advanced Materials (RIAM), Seoul National University, Seoul 08826, South Korea; Gyeongbuk Science Technology Promotion Center, Gumi Electronics & Information Technology Research Institute, Gumi 39171, South Korea; Stuttgart Center for Electron Microscopy, Max Planck Institute for Solid State Research, Heisenbergstrasse 1, 70569 Stuttgart, Germany

**Keywords:** Nanoparticle, Electron-beam irradiation, Transmission electron microscopy

## Abstract

As is well documented, platinum nanoparticles, promising for catalysts for fuel cells, exhibit better catalytic activities, when alloyed with Zn. Pre-existing syntheses of Pt–Zn alloy catalysts are composed of a number of complex steps. In this study, we have demonstrated that nanoparticles of Pt–Zn alloys are simply generated by electron-beam irradiation in a transmission electron microscope of a wurtzite ZnO single-crystal specimen. The initial ZnO specimen is considered to have been contaminated by Pt during specimen preparation by focused ion beam milling. The formation of the nanoparticle is explained within the framework of ionization damage (radiolysis) by electron-beam irradiation and accompanying electrostatic charging.

## Background

Platinum is one of the best noble metals as a catalyst for methanol steam reforming to produce hydrogen in indirect methanol fuel cells [1]. Interestingly, for Pt, the methanol conversion is reported to be increased by forming an alloy with Zn [2–5]. Furthermore, Pt–Zn alloy catalysts exhibit higher selectivity to carbon dioxide (CO_2_), rather than carbon monoxide (CO) [2, 3, 5]. Also for direct use of liquid fuels, such as methanol and formic acid (i.e., for direct methanol and formic acid fuel cells), it acts as one of the best catalysts for electro-oxidation of the fuels [6, 7]. However, Pt is prone to poisoning by CO [8–11], which reduces its catalytic performances and thus prevents it from being widely used. The problem has been partly resolved by syntheses of Pt-based alloys or intermetallic compounds (e.g., Pt–Zn), which exhibit higher CO poisoning tolerance [12, 13]. Nanoparticles (NPs) of Pt–Zn alloys were observed to prepare, for example, by reducing ZnO in the case of Pt salts impregnated onto a ZnO support [2], by addition of a Zn nitrate as a promoter to an aqueous solution of Pt salts [3], or by preparing Pt nanoparticles with a ZnO shell [5]. However, these processes are composed of complicated wet chemical syntheses and post heat-treatments [1–13]. It would be a great technological improvement if a strategy to shorten the previous, lengthy processes is provided.

In the present study, we introduce a simple, interesting synthesis of Pt–Zn alloy NPs. We demonstrate that such alloy particles can be formed by electron-beam irradiation in a transmission electron microscope (TEM) of a wurtzite ZnO single-crystal specimen. ZnO crystallizes in either a wurtzite (hexagonal), a zincblende (cubic), or a rocksalt (cubic) structure [14, 15]. Unless stated otherwise, “ZnO” in the main text henceforth means the wurtzite phase. The initial ZnO specimen used in the present study is considered to have been contaminated by Pt during specimen preparation for TEM by focused ion beam (FIB) milling. The NPs are identified to be a Pt–Zn alloy with a face-centered cubic (fcc) structure by high-resolution TEM (HRTEM), energy dispersive X-ray spectroscopy (EDXS), and electron energy low spectroscopy (EELS). It is suggested that the formation of the Pt–Zn NPs occurs by ionization damage (radiolysis) by electron-beam irradiation and accompanying electrostatic charging [16–23].

## Methods

**Fig. 1:**
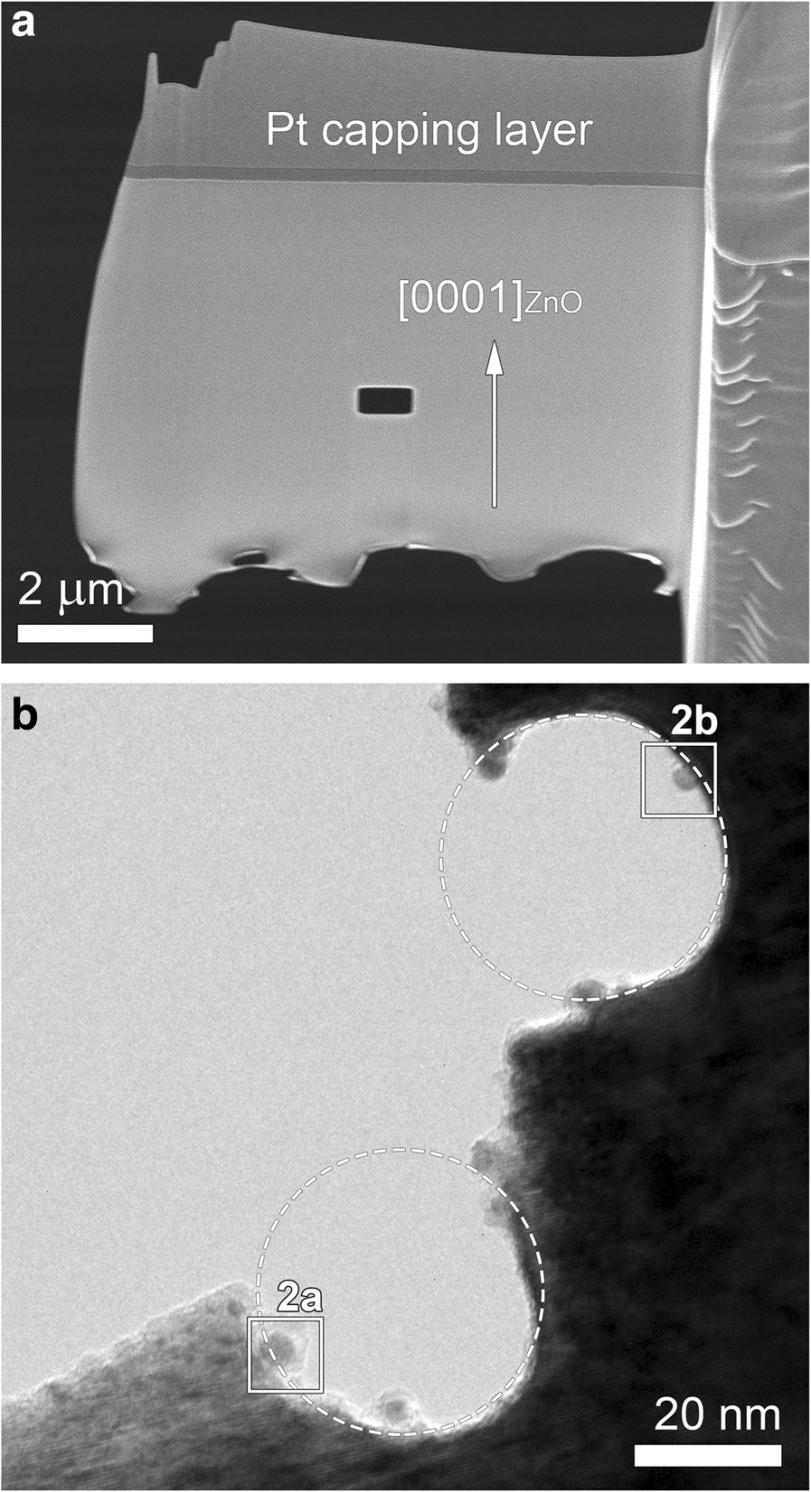
**a** SEM microstructure of a FIB lamella. A rectangular hole is intentionally drilled in the middle of the specimen during FIB to obtain a uniformly thin region for observation. The region around the hole is beam-irradiated. **b** Low-magnification TEM image showing that two holes are formed after beam irradiation by focusing an electron beam in a diameter of 40 nm for 105 s, as indicated by *circles*. Nanoparticles are observed to protrude toward the center of the holes formed by beam irradiation

In this study, we used a nominally undoped ZnO single crystal with a surface normal direction of [0001] (MaTecK GmbH) for TEM. Specimens for TEM were prepared from the single crystal on a focused Ga-ion beam (FIB) workstation (FEI Nova 200 Nanolab DualBeam FIB, FEI). The surface of the crystal was coated by Pt for scanning electron microscopy, which was followed by the deposition of a protective Pt capping layer for FIB. A cross-section lamella was lifted out and attached to a Mo half-grid. Ga-ion milling was performed at 5–30 kV. We intentionally drilled a rectangular hole in the middle of the lamella during FIB (Fig. 1a), which was effective to get a uniformly thin region without a thick amorphous layer (see the bottom edge of the specimen, which was rugged and severely warped). Low kV thinning and cleaning were finally carried out at 5 kV with small currents (a few tens of picoamperes) on both sides of the specimen.

Electron irradiation experiments were done in the TEM mode of a field emission TEM (JEOL JEM-2100 F, operated at 200 kV) by focusing an electron beam with a current of 7 nA in diameters ranging from 14 to 50 nm onto the specimen, resulting in current densities at the specimen of 4550 down to 360 A/cm^2^. All HRTEM images were taken along the zone axis of [1 –2 1 0]. The current density at the specimen during observation was in the range of 15–20 A/cm^2^. The base pressure in the specimen chamber of the JEM 2100F was 1–3 × 10^–5^ Pa. The irradiated specimens were examined using the JEM-2100F for HRTEM imaging.

Composition analyses of local areas of interest were performed by EDXS with a microanalysis system (AZtec, Oxford Instruments) in a Cs-corrected STEM (JEOL JEM-ARM-200F). The ARM-200F is operated at 200 kV and equipped with a spherical aberration corrector (CEOS GmbH). The STEM-EDXS was operated at a probe diameter of <0.1 nm in the spectrum imaging (SI) mode. The SI mode produces a two-dimensional array of pixels, each pixel that contains a full X-ray spectrum, allowing quantitative mapping of materials’ chemistry. Using SI, specimen drift can be compensated to a certain degree and electron-beam damage during EDXS is minimized. As needed, we could reconstruct spectra from specific positions in a mapping image by area selection. We set in AZtec software the sigma level to 3, which corresponds to the three-sigma confidence level (99.7 %) for a normal statistical error distribution. Also for EELS of NPs, rather than relying on point analysis, we performed SI for the same reasons as for EDXS. EELS measurements were done with a Gatan GIF Quantum ER system attached to JEOL JEM-ARM-200F. The electron probe diameter is about 0.25 nm. The energy resolution at the zero-loss peak is typically 0.5 eV. The EELS spectra were acquired in the SI mode using a 2.5-mm EELS entrance aperture, which determined the collection semiangle of 41.7 mrad at a dispersion of 0.05 eV per channel.

## Results

As specified in the “Methods” section above, a rectangular hole was intentionally drilled in the middle of the specimen during FIB to get a uniformly thin region (Fig. 1a). We irradiated the thin region by focusing an electron beam in various diameters (see Methods). Figure 1b shows a low-magnification TEM image of a thin region around the hole irradiated by focusing an electron beam in a diameter of ~40 nm (corresponding to a current density of ~560 A/cm^2^) for 105 s. As indicated by white dashed circles, holes were drilled by beam irradiation, and interestingly, NPs are observed to protrude toward the inside of the holes (Fig. 1b).

**Fig. 2:**
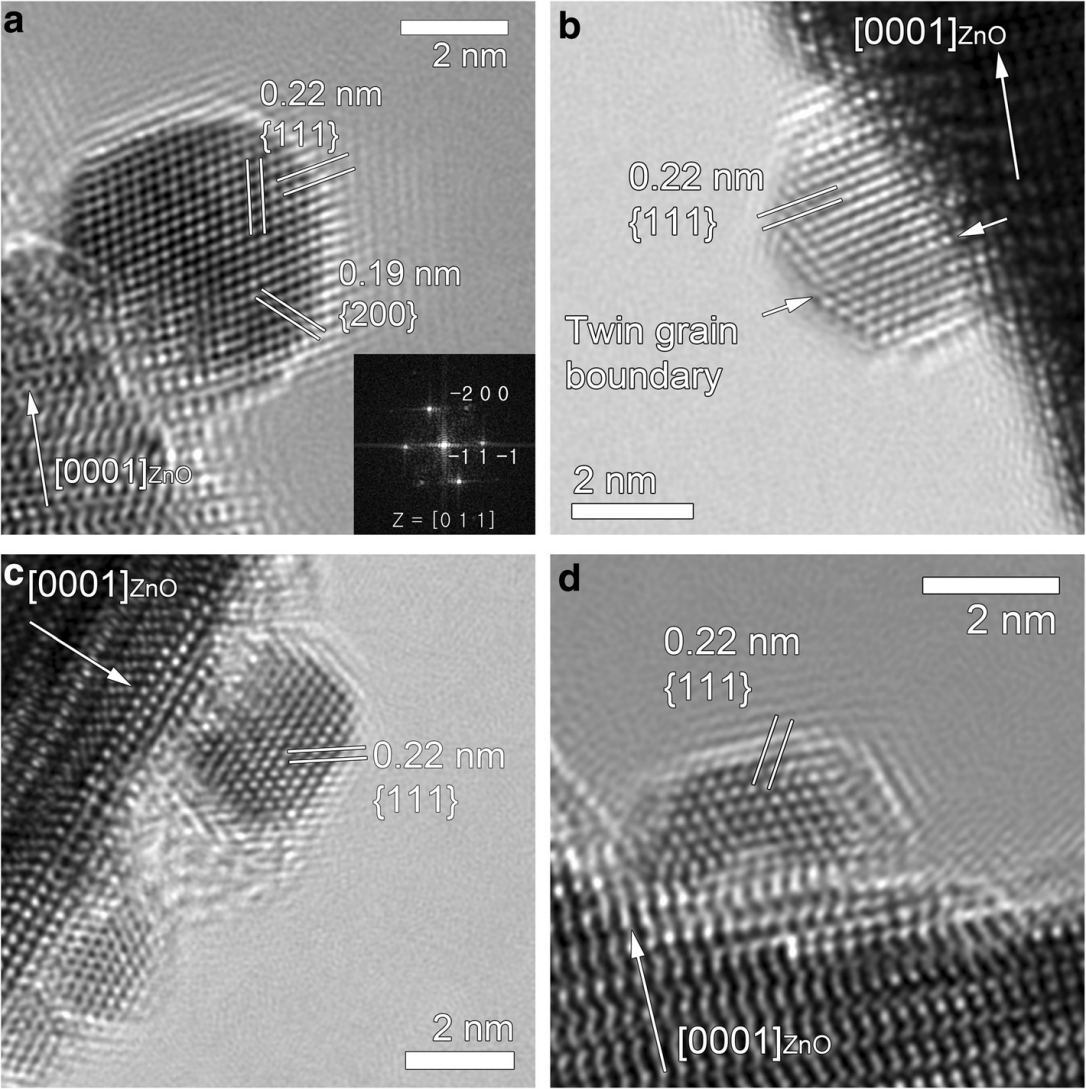
Morphology and crystal structure of NPs. **a** Higher magnification of the boxed region marked as 2a in Fig. 1b and a Fourier transform of the NP (*inset*). **b** Higher magnification of the boxed region marked as 2b in Fig. 1b. HRTEM images of NPs formed by focusing an electron beam in diameters of **c** 50 nm for 120 s and **d** 14 nm for 30 s. All the NPs observed show an fcc structure with an estimated lattice parameter of ~0.38 nm. See text for details

An enlarged view of a boxed region marked as 2a in Fig. 1b (Fig. 2a) shows a NP. The crystal structure of the right particle is different from that of the ZnO matrix. The interplanar spacings for two types of planes are measured to be 0.22 and 0.19 nm, the ratio of the two values being 1.16, and the angle between the two planes is ~55^°^. The values agree with those from a cubic structure, where the ratio of the interplanar spacing for the {111} planes to that for the {002} planes is 1.155 and the interplanar angle is 54.74°. The Fourier transform of the NP (Fig. 2a, inset) indicates that the crystal has an fcc structure, its lattice parameter (*a*) being thus estimated to be ~0.38 nm. The relevant planes are indexed in Fig. 2a. Such NPs are typically observed around the hole formed by beam irradiation (Fig. 2b–d). Figure 2b shows a magnified, HRTEM image of a boxed region (2b) in Fig. 1b. This NP also shows a similar structure to that in Fig. 2a, and in this case, is twinned. NPs shown in Fig. 2c, d, which are obtained by focusing an electron beam in diameters of ~50 nm (corresponding to a current density of ~360 A/cm^2^) for 120 s and ~14 nm (corresponding to a current density of ~4550 A/cm^2^) for 30 s, respectively, also reveal similar structures to those in Fig. 2a, b. All the NPs observed (Fig. 2) are observed to be constantly shaking during observation.

**Fig. 3:**
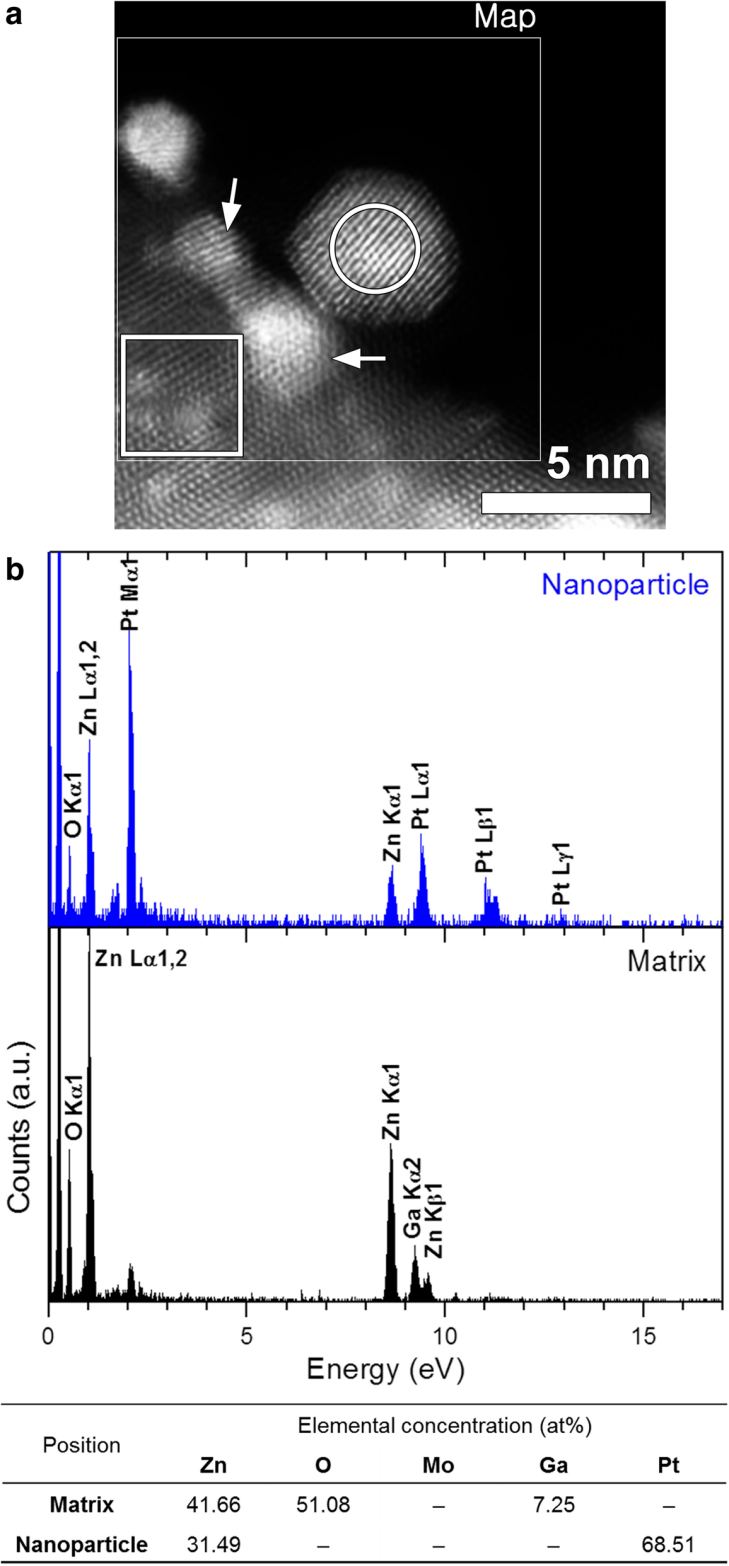
**a** HAADF-STEM image of the NP shown in Fig. 2a and its surroundings. **b** EDXS spectra acquired over the NP and matrix regions marked by a *circle* and a *square*, respectively, in **a**. During EDXS mapping, the used pixel dwell time was ~4.4 ms at a spatial dimension of ~0.04 nm/pixel

**Fig. 4:**
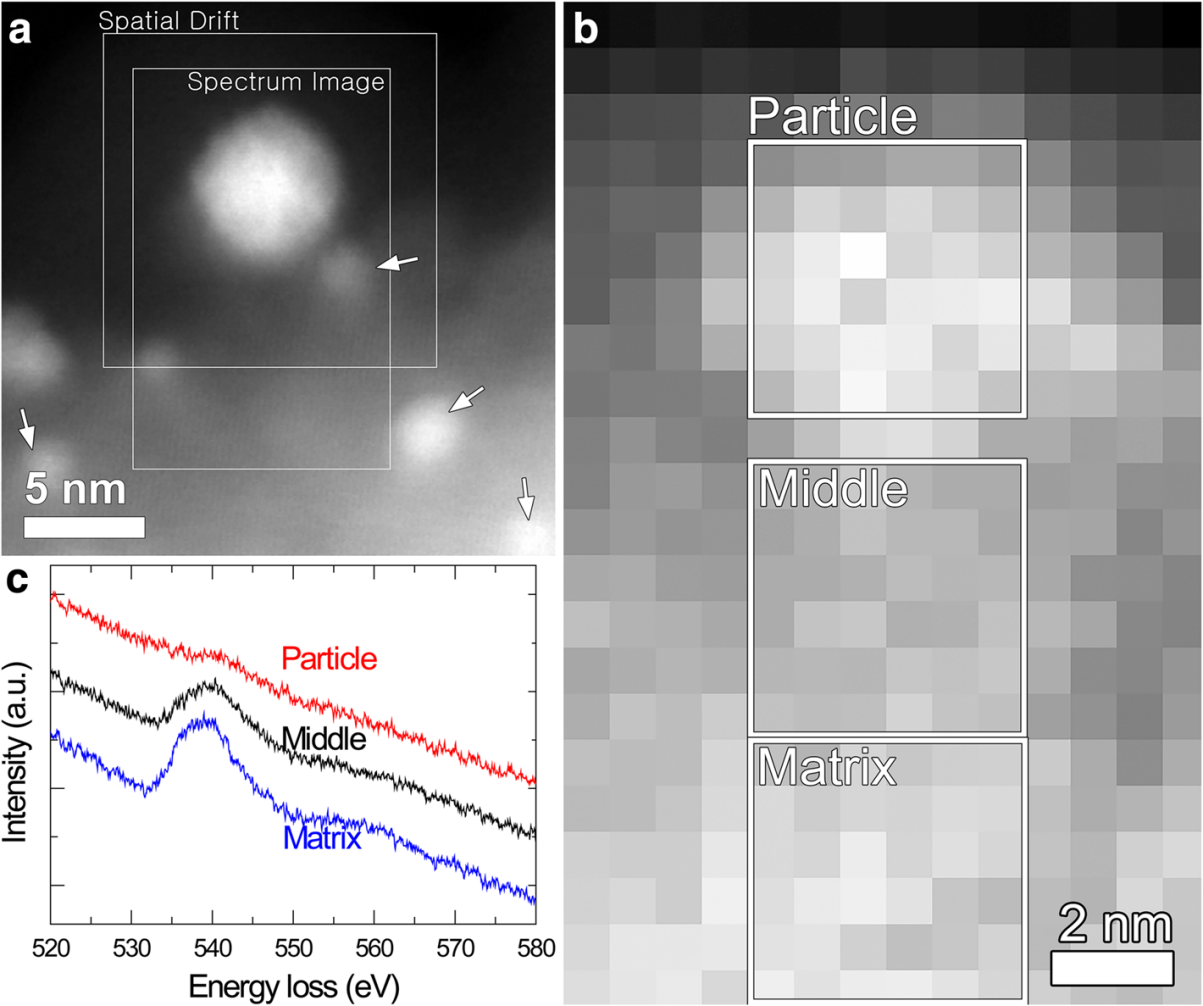
**a** HAADF-STEM image of a NP and its surroundings. **b** EEL spectrum image for the O-K edge (532 eV) acquired over a region including the NP framed in Fig. 4a. Dimensions of the scanned area were 10.65 × 16.73 nm^2^, corresponding to a spectrum image with 14 × 22 pixels of 0.76 × 0.76 nm^2^ pixel size. The dwell time per pixel was 1 s. The total acquisition time including a process time was 12 min 23 s. The aforementioned dwell time was determined to minimize electron-beam damage of the NP during EELS. **c** Separate EEL spectra integrated over the *white framed regions* in **b**

The chemical composition of NPs was analyzed by scanning TEM (STEM)-EDXS in the spectrum imaging (SI) mode (Fig. 3). Figure 3 shows a high-angle annular dark field (HAADF)-STEM image of the NP shown in Fig. 2a and EDXS spectra acquired over the NP and matrix regions. As represented in Fig. 3, the NPs observed in the present study are determined to be Pt–Zn alloys. The HAADF-STEM image (Fig. 3a) shows that there are bright spots in the matrix region, some of which are indicated by white arrows. These spots are identified to be due to Ga contamination, which is not seen in the HRTEM images (Fig. 2). The Ga contamination is reflected in the composition table (Fig. 3b). The EDXS result (Fig. 3) showing that the particle did not contain any oxygen is supported by EELS, as shown in Fig. 4. Figure 4a, b presents a HAADF-STEM image of a NP and its surroundings and the corresponding spectrum image for the O-K edge (532 eV). There exists a very weak O-K edge signal appearing at 532 eV for the NP examined (Fig. 4c), probably originating from oxygen on the NP surface. Bright spots in the matrix region as indicated by white arrows in Fig. 4a are due to Ga contamination, as noted above. Note that Pt was not detected in the un-irradiated matrix (Fig. 3b).

All the observations taken together, the NPs are Pt–Zn alloys (Figs. 3 and 4) and their lattice parameters are measured from the HRTEM images (Fig. 2) to be smaller than that of Pt (fcc (space group: $Fm\overline{3}m$), *a* = 0.392 nm). Thus, we conclude that Zn atoms with an atomic radius (142 pm) smaller than that of Pt (177 pm) [24] were successfully incorporated into Pt fcc structure, forming Pt–Zn alloys with contracted lattices.

## Discussion

Electron-beam damages in the TEM for inorganic specimens are largely classified into knock-on damage which means atomic displacement by the electron beam through elastic scattering and radiolysis which indicates chemical bond breaking through inelastic scattering (ionization) [16, 25, 26]. Knock-on atomic displacement occurs only above some threshold incident electron energies, and radiolysis can occur even below threshold values. For ZnO, the displacement energy is taken to be 18.5 and 41.4 eV for Zn and O, respectively [27], corresponding to displacement thresholds [16] of 398 and 244 keV, respectively. These threshold values are above the acceleration voltage (200 keV) in the present study, thus which leads us to exclude the possibility that knock-on damage induces the hole drilling and the formation of the new phases (Figs. 1 and 2).

The hole drilling observed in the present study might result from surface sputtering, because the process can occur at voltages of 50 % or less of knock-on thresholds [28]. However, generally cross sections for knock-on displacement as well as surface sputtering are lower by far than those for radiolysis [29], and effects of radiolysis would be major for insulating materials like ZnO.

Under electron-beam irradiation in the TEM of an insulating inorganic specimen, radiolysis by inelastic scattering occurs close to the specimen surface [16, 19–22]. During irradiation, few incident electrons are absorbed in the case of a thin specimen in the TEM. Even more electrons than supplied by the incident electrons are emitted into the vacuum and the surrounding region as a result of backscattering, Auger, and secondary electron emissions, which make the specimen surface positively charged for a resistive specimen like ZnO [16–23]. Positive charging is supposed to drive atoms of an inorganic solid to be ionized, which leads the solid to decompose by electrostatic repulsion between its ionized constituent atoms, eventually forming a hole in the specimen, as shown by Humphreys et al. [22] and in the present study.

Radiolysis in transition-metal oxides is suggested to occur via the Knotek-Feibelman mechanism [30, 31], which indicates that an oxygen anion become positively charged by interatomic Auger decays of the core holes. Resultantly, the positively charged oxygen ions are repelled by metal cations into the surrounding vacuum or the un-irradiated neighboring region. Applying the mechanism to the present system, the irradiated area of ZnO is considered to be decomposed into Zn cations and positive oxygen ions and eventually becomes perforated by electrostatic repulsion between these positive ions.

**Fig. 5:**
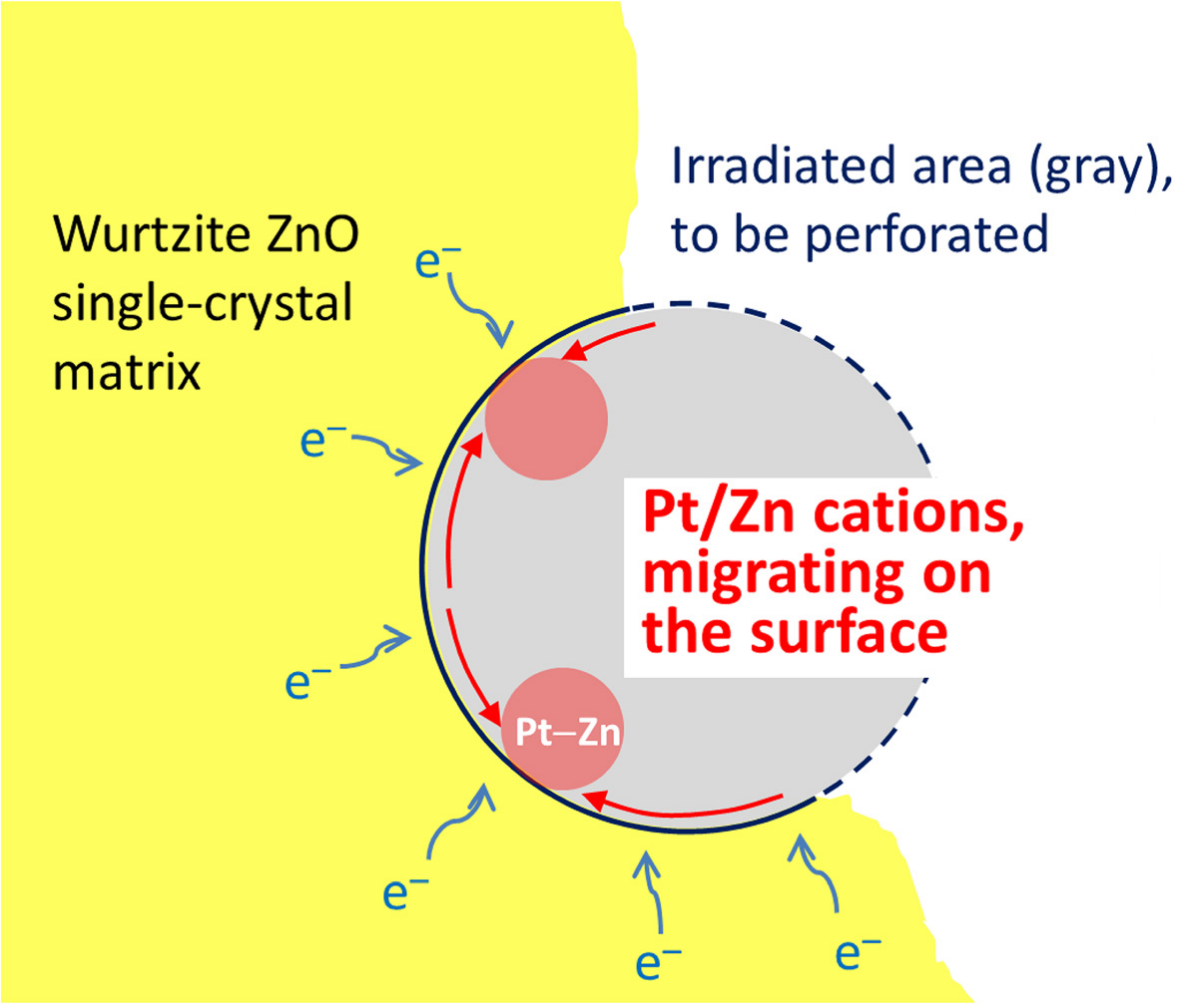
Schematic diagram showing the formation of Pt–Zn alloy NPs. The formation of the Pt–Zn alloy NPs seems to be related to the atomic weights (or relative atomic masses) of these ions. The atomic weights of Pt (195.078) and Zn (65.409) are larger than that of O (16). Since the Pt and Zn ions are heavier than the O ions, the metal ions are more probable to stay longer around the hole, resisting electrostatic repulsion. By accepting specimen electrons from the surroundings, a current of electrons that is induced by the positive charging of the irradiated region to achieve current balance, they can form Pt–Zn alloy NPs with a higher fraction of Pt

The formation of the Pt–Zn alloy also seems explained within the framework of radiolysis and accompanying electrostatic charging. First, we should address where Pt comes from. Since the ZnO crystal used in the present study did not contain any Pt, Pt atoms are considered to originate from the Pt deposition and capping layer deposited before TEM specimen preparation by FIB milling (Fig. 1a). During FIB milling and cleaning, the specimen could be thus contaminated by Pt. As noted above, the ZnO matrix in the irradiated region undergoes the dissociation into Zn and O positive ions by radiolysis. Under beam irradiation, Pt impurity atoms would also become positively charged, because they are electrically isolated on the resistive ZnO. Those breakaway Zn, O, and Pt positive ions are likely to be desorbed into the vacuum under electrostatic charging. The formation of the Pt–Zn alloy NPs suggests that some Pt and Zn ions could linger or migrate on the surface of the hole without being desorbed into the vacuum. The phenomenon seems related to the atomic weights (or relative atomic masses) of these ions. The atomic weights of Pt (195.078) and Zn (65.409) are larger than that of O (16). Since the Pt and Zn ions are heavier than the O ions, the metal ions are more probable to linger longer around the hole, resisting the repulsive force. And they will accept specimen electrons from the surroundings, a flow of electrons that is attracted into the irradiated region by the positive charging there to achieve current balance [16, 21, 23]. These processes will lead to the formation of Pt–Zn alloy NPs with a higher fraction of Pt, as manifested by Fig. 3. The composition of the NPs roughly approximates to that of Pt_3_Zn, which is an ordered intermetallic compound with a prototype of AuCu_3_ (space group: $Pm\overline{3}m$, *a* = 0.3893 nm [32]), but as exemplified in Fig. 2a, the particles just have a disordered AuCu_3_ structure, which is because they were not thermally activated enough. The aforementioned mechanism of the formation of the Pt–Zn NPs is illustrated in Fig. 5. Arrows indicate the direction of specimen electrons toward the positively charged region marked by a gray-shaded circular area.

Recently, in another independent beam-irradiation experiment, we observed a rare formation of ZnO NPs with a rocksalt structure, which is known to be a high-pressure phase, just by beam irradiation without any pressure applied [33]. The rocksalt ZnO NPs formed around the hole drilled by beam irradiation, as for the Pt–Zn alloy NPs in the present study. We should address where the difference stems from. We find that, if a specimen is cleaned further at a lower voltage during FIB (i.e., 2 kV for the previous study [33]), no Pt–Zn alloy NPs form. I.e., further cleaning would remove the source of Pt, which could form Pt–Zn alloy phases, and instead, lead to a rare formation of rocksalt ZnO NPs, as observed in the previous work [34]. Their formation was explained by size effect, as suggested by Koster et al. [34]. Using first-principles calculations, they claim that there is a critical size, below which the total energy of rocksalt ZnO becomes smaller than that of wurtzite ZnO [34]. Put it another way, Pt impurity atoms, if remaining in a specimen, are considered to incorporate Zn atoms into their lattice, inhibiting the possible formation of rocksalt ZnO NPs. This is the reason why rocksalt ZnO NPs are not observed in the present study.

## Conclusions

We have fabricated NPs of Pt–Zn alloys by focusing an electron beam on a ZnO single crystal in the TEM. The initial ZnO specimen is considered to have been contaminated by Pt during TEM specimen preparation by FIB milling. The phenomenon is interpreted as resulting from radiolysis and positive charging of the specimen under electron-beam irradiation. The present results await further study to reproduce the synthesis of the Pt–Zn alloy NPs in equipment outside the TEM (e.g., an electron lithography system) for use as catalysts in fuel cells.

## Abbreviations

EDXS, energy dispersive X-ray spectroscopy; EELS, electron energy loss spectroscopy; FIB, focused Ga-ion beam; HAADF, high-angle annular dark field; HRTEM, high-resolution TEM; NP, nanoparticle; SI, spectrum imaging; STEM, scanning TEM; TEM, transmission electron microscopy
